# A midposition NOTCH3 truncation in inherited cerebral small vessel disease may affect the protein interactome

**DOI:** 10.1016/j.jbc.2022.102772

**Published:** 2022-12-05

**Authors:** Soo Jung Lee, Xiaojie Zhang, Gang Xu, Jimo Borjigin, Michael M. Wang

**Affiliations:** 1 Department of Neurology, University of Michigan, Ann Arbor, Michigan, USA; 2 Neurology Service, VA Ann Arbor Healthcare System, Ann Arbor, Michigan, USA; 3 Molecular and Integrative Physiology, University of Michigan, Ann Arbor, Michigan, USA

**Keywords:** NOTCH3, CADASIL, Asp-Pro bond, nonenzymatic cleavage, protein interactions, CADASIL, cerebral autosomal dominant arteriopathy with subcortical infarcts and leukoencephalopathy, cDNA, complementary DNA, EGF, epidermal growth factor, IHC, immunohistochemical, TBST, Tris-buffered saline with Tween-20, TCEP, Tris(2-carboxyethyl)phosphine

## Abstract

Mutations in *NOTCH3* underlie cerebral autosomal dominant arteriopathy with subcortical infarcts and leukoencephalopathy (CADASIL), the most common inherited cerebral small vessel disease. Two cleavages of NOTCH3 protein, at Asp80 and Asp121, were previously described in CADASIL pathological samples. Using monoclonal antibodies developed against a NOTCH3 neoepitope, we identified a third cleavage at Asp964 between an Asp-Pro sequence. We characterized the structural requirements for proteolysis at Asp964 and the vascular distribution of the cleavage event. A proteome-wide analysis was performed to find proteins that interact with the cleavage product. Finally, we investigated the biochemical determinants of this third cleavage event. Cleavage at Asp964 was critically dependent on the proline adjacent to the aspartate residue. In addition, the cleavage product was highly enriched in CADASIL brain tissue and localized to the media of degenerating arteries, where it deposited with the two additional NOTCH3 cleavage products. Recombinant NOTCH3 terminating at Asp964 was used to probe protein microarrays. We identified multiple molecules that bound to the cleaved NOTCH3 more than to uncleaved protein, suggesting that cleavage may alter the local protein interactome within disease-affected blood vessels. The cleavage of purified NOTCH3 protein at Asp964 *in vitro* was activated by reducing agents and NOTCH3 protein; cleavage was inhibited by specific dicarboxylic acids, as seen with cleavage at Asp80 and Asp121. Overall, we propose homologous redox-driven Asp-Pro cleavages and alterations in protein interactions as potential mechanisms in inherited small vessel disease; similarities in protein cleavage characteristics may indicate common biochemical modulators of pathological NOTCH3 processing.

*NOTCH3* mutations result in cerebral autosomal dominant arteriopathy with subcortical infarcts and leukoencephalopathy (CADASIL) (1), the most common inherited cerebral small vessel disease (2, 3). The overwhelming majority of CADASIL mutations affect the number of cysteines in the NOTCH3 gene product (4, 5), a characteristic that is capable of affecting one or more of the 102 disulfide bonds of the epidermal growth factor (EGF) domain array region of the extracellular domain of the protein.

Pathological examination of CADASIL reveals marked accumulation of NOTCH3 protein (6) and other matrix proteins (7, 8, 9, 10, 11) in the smooth muscle layer of cerebral arteries. Protein accumulation is accompanied by severe vascular smooth muscle degeneration (12). These genetic and pathological characteristics of CADASIL are consistent with a neomorphic mechanism of disease in which alterations of NOTCH3 tertiary structure trigger protein accumulation and vascular smooth muscle toxicity.

The structural changes in NOTCH3 that occur in CADASIL have been revealed in studies that compare purified wildtype and mutant proteins (13, 14). In contrast to normal NOTCH3, CADASIL mutants oligomerize, harbor free thiol groups, fragment readily, and are vulnerable to transreduction *via* cysteine containing NOTCH3 fragments (13, 14, 15, 16).

Additional modifications of NOTCH3 also include a series of post-translational alterations of NOTCH3: (a) redox-dependent changes in structure (17) and (b) pathological cleavage of the protein (18, 19). Two cleavages of NOTCH3 have been identified so far: cutting C terminal to Asp80 (18) and Asp121 (19). The cleavage of NOTCH3 at these locations is predicted to release two N-terminal NOTCH3 fragments (NTF and NTF2), which can bind to intact NOTCH3 ectodomain and transreduce a number of vascular matrix proteins (13, 15, 19). Both these cleavage events occur at Asp-Pro bonds situated between adjacent EGF-like domains (between repeats 1 and 2 for Asp80 and between 2 and 3 for Asp121). Four additional Asp-Pro sequences are found in human NOTCH3 ectodomain and have not yet been characterized.

In the current work, we examined a third Asp-Pro site in NOTCH3 as a potential target of post-translational proteolysis in CADASIL. Using monoclonal antibodies that bind to the neoepitope generated by cleavage at Asp964, we describe the structural determinants and histological localization of this cleavage event. To understand potential consequences of the cleavage, we performed proteome-wide analysis of molecules capable of interacting with the product of Asp964 proteolysis. Finally, *in vitro* studies identified inhibitors and activators of cleavage, which suggest a mechanism that parallels that of cleavage at Asp80 and Asp121.

## Results

### Asp-Pro sequences in human NOTCH3

**Figure 1:**
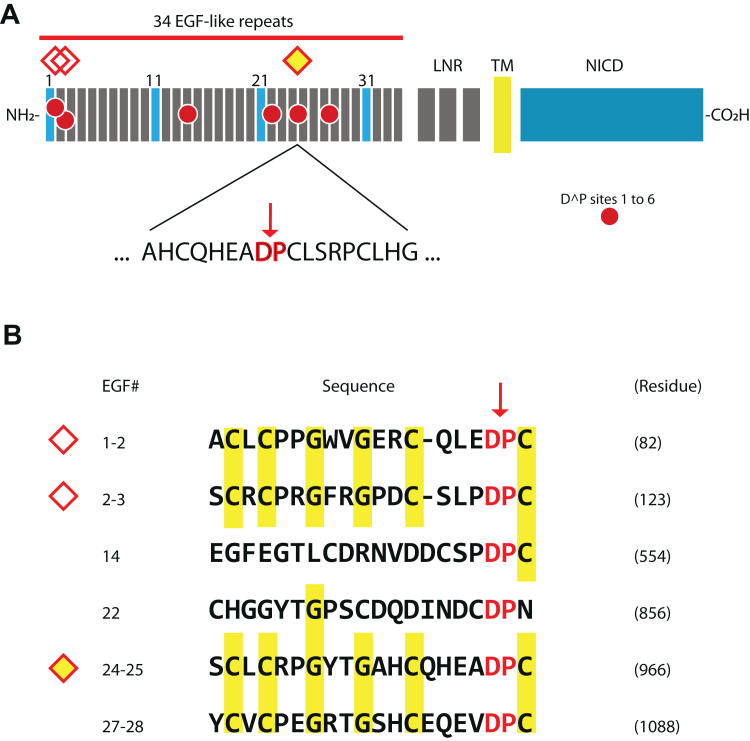
**Asp-Pro sequences in NOTCH3.***A*, human NOTCH3 is composed of 34 tandem EGF-like repeats and LNR sequences, which compose the ectodomain of the protein (to the *left* of the transmembrane [TM] sequence). The Notch intracellular domain (NICD) is at the carboxyl terminus of the protein. *Red circles* depict the six Asp-Pro sites within EGF-like repeats. The *open diamonds* show Asp-Pro cleavage sites that have been previously characterized in CADASIL. The *yellow diamond* at the border between EGF24 and EGF25 is the focus of this work; the border sequence is shown below the protein schematic with an *arrow* highlighting the Asp-Pro junction. *B*, a comparison of the sequences N-terminal to the six Asp-Pro sequences is shown. Conserved sequences that are common to the previously described cleaved Asp-Pro regions (top two sequences) are highlighted in *yellow*. The third and fourth sequences are contained with EGF14 and EGF22 and are not at borders between two EGF-like domains. The focus of this work is represented by the *yellow diamond*. Residue number of the most C-terminal amino acid of each sequence is shown for reference. CADASIL, cerebral autosomal dominant arteriopathy with subcortical infarcts and leukoencephalopathy; EGF, epidermal growth factor.

There are six Asp-Pro sequences in the EGF-like repeat domain of human NOTCH3. Two sequences at positions 80 to 81 and 121 to 122 have been described before as disease-related sites of proteolysis in CADASIL (18, 19). Additional sites at positions 552 to 553, 854 to 855, 964 to 965, and 1086 to 1087 are located within the EGF-like repeats (Fig. 1*A*). Sites at positions 80 to 81, 121 to 122, 964 to 965, and 1086 to 1087 are situated between EGF-like repeats. Although all these Asp-Pro sequences may be candidate cleavage sites, this study focuses on sequences 964 to 965, which we hypothesized could be cleaved based on similarity to 80 to 81 and 121 to 122 (Fig. 1*B*). In prior work, we described generation of antibodies that recognize a neoepitope specific for the cleaved protein and that requires liberation of the C-terminal aspartic acid residue at the site of proteolysis (18, 19). A similar approach was taken in this work in which we generated neoepitope antibodies to detect cleavage of NOTCH3 at Asp964.

### Generation of monoclonal antibodies against a NOTCH3 peptide ending at Asp964

Rabbit monoclonal antibodies were generated against a peptide found in NOTCH3 ending at Asp964: SCLCRPGYTGAHCQHEAD. Several clones were obtained with high specificity for the peptide. Clone 69B was selected for further characterization and used for all experiments described later.

**Figure 2:**
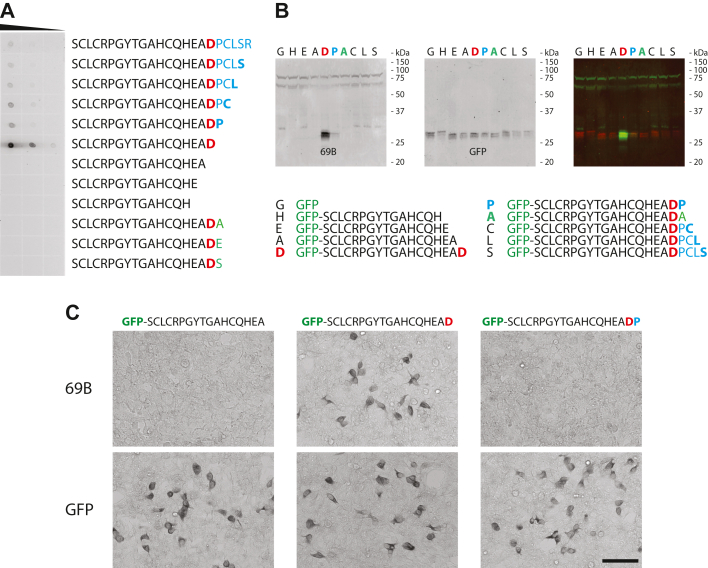
**Epitope characterization for antibody 69B.** The specificity of antibody 69B was determined by dot blotting (*A*) and Western blotting (*B*). *A*, dot blots that included synthetic peptides based on the predicted neoepitope generated by Asp964 cleavage of NOTCH3 were probed with 69B. The peptide sequences are shown on the *right side*; all amino acids are present in the native protein except for the last three sequences that are substitutions of proline normally present at residue 965. The neoepitope expected from Asp964 cleavage is SCLCRPGYTGAHCQHEAD. Peptides were serially diluted at 100, 10, and 1.0 μg/ml, and 1 μl was spotted per dot. *B*, immunoblots of recombinant GFP fused to NOTCH3 sequences were probed with 69B or with GFP. The merged image of both antibody probes is shown in *color*. The GFP sequences were appended with the sequences shown at the *bottom* and include native NOTCH3 sequences. Corresponding *single letter codes* for each sequence are shown above each lane of the blots. *C*, human embryonic kidney (HEK)293 cells were transfected with indicated constructs and then immunostained. We show results from staining with 69B (*top row*) or GFP antibodies (*bottom row*). Scale bar respresents 100 microns.

When tested against peptides on dot blots, 69B demonstrated a strong preference for sequences that ended precisely with the immunogen (Fig. 2*A*). The antibody did not recognize peptides with deletions of the terminal aspartate residue or peptides that contained addition of non-native residues after Asp964. There was very weak binding to peptides, which contained amino acid extensions beyond the aspartate of the immunogen.

To test the requirement for the C-terminal aspartic acid residue for antibody recognition on immunoblots, we generated recombinant constructs that appended the target sequence to the C terminus of GFP. Additional constructs featured deletions and extensions of amino acids to the sequence ending in Asp964 of NOTCH3. When proteins from cells transfected with these constructs were evaluated by immunoblotting using 69B, only the protein ending precisely with Asp964 was strongly recognized (Fig. 2*B*).

Cells transfected with selected GFP constructs in Figure 2*B* were fixed and stained using 69B. Though all transfectants stained equally for GFP, only cells receiving constructs that ended precisely with terminal Asp964 showed significant staining with 69B; deletion of the terminal Asp964 residue and addition of Pro965 to the recombinant GFP eliminated cell staining (Fig. 2*C*). We concluded that overall, 69B appears to strongly recognize protein ending in Asp964 of NOTCH3.

### Susceptibility of NOTCH3 to cleavage at Asp964

**Figure 3:**
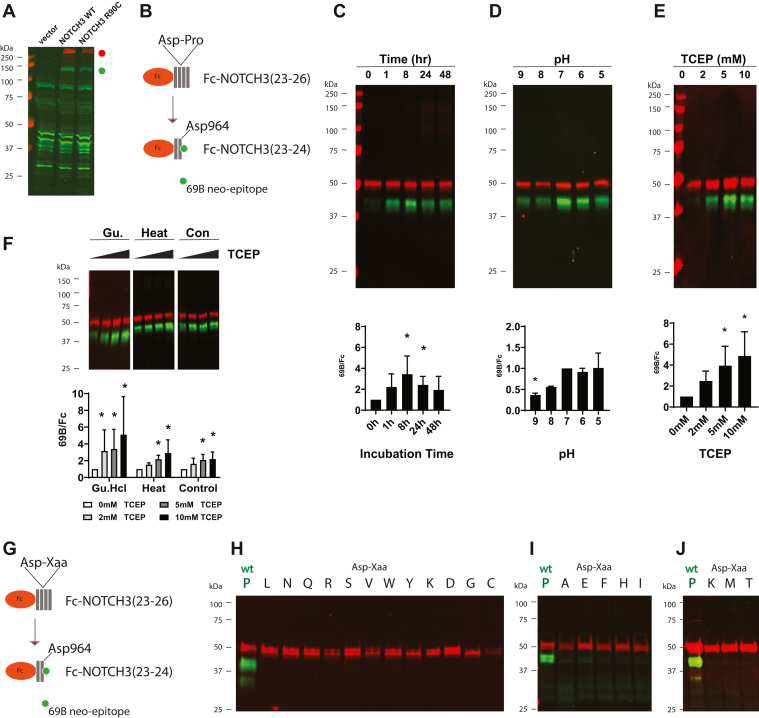
**Cleavage of recombinant NOTCH3 at Asp964.** Western blots of purified recombinant NOTCH3 protein were performed with 69B to detect fragmentation of protein at Asp964. *A*, lysates from 293 cell transfected with vector, wildtype (WT) full-length human NOTCH3 complementary DNA (cDNA), or the CADASIL mutant R90C were incubated for 1 h in radioimmunoprecipitation assay (RIPA) plus 5 mM TCEP and compared by immunoblotting using 69B (*green*) and an antibody to N-terminal NOTCH3 (M01, Abnova, *red*). *B*, to study purified NOTCH3 fragmentation, fragments (EGF domains 23–26) fused to Fc (Fc-NOTCH3(23–26)) were studied. Schematic showing purified protein used to detect spontaneous cleavage at Asp964, situated at the junction between NOTCH3 EGF-like repeats 24 and 25. *C*, to initiate cleavage, purified Fc-NOTCH3(23–26) was diluted at 10 μg/ml in water and incubated at 37 °C for periods indicated. Proteins were separated by SDS-PAGE, transferred to nitrocellulose, and then probed with 69B (*green bands*) and secondary antibodies to mouse Fc (*red bands*). Quantification shows the ratio of 69B intensity to Fc intensity. *D*, the same protein was diluted at pH specified and similarly incubated and analyzed. Reaction time was 1 h. *E*, the same protein was diluted with water and TCEP at the final concentrations shown prior to quantification of cleavage by measurement of the ratio of 69B intensity to Fc intensity. *F*, Fc-NOTCH3(23–26) was pretreated with guanidinium hydrochloride (1 M) or heat (65 °C for 60 min) or untreated; this was followed by treatment with TCEP as indicated to stimulate cleavage. Analysis was performed to characterize Asp964 cutting as above. *G*, the construct encoding Fc-NOTCH3(23–26) was subjected to site-directed mutagenesis at Pro965 (WT sequence) to all other amino acids. Each resulting mutant was transiently transfected into 293 cells, and protein from the conditioned media was concentrated with Protein A-agarose. The captured protein was analyzed as in (*A*) to quantify the amount of cleavage at Asp964 (*H*–*J*). Please note that the lysine (K; P965K) mutant appears twice; three gels were run to display the cleavage of all mutants relative to the WT sequence. Experiments were performed at least three times. Merged pseudocolored images are shown, with *green* for 69B and *red* for Fc. ∗ indicates differences between group with a *p* < 0.05. CADASIL, cerebral autosomal dominant arteriopathy with subcortical infarcts and leukoencephalopathy; EGF, epidermal growth factor; TCEP, Tris(2-carboxyethyl)phosphine.

Transfected 293 cells were analyzed to assess if NOTCH3 is cleaved in cells. Vector-transfected, wildtype full-length human NOTCH3, and R90C mutant–transfected cell lysates were incubated with 5 mM Tris(2-carboxyethyl)phosphine (TCEP) and analyzed by immunoblotting (Fig. 3*A*). Only NOTCH3-transfected cells expressed full-length NOTCH3 (*red dot*); this band did not react with 69B. Furthermore, using 69B, a faster migrating band appeared that was unique to NOTCH3-transfected cells. This finding was consistent with cleavage at Asp964 of the full-length wildtype and mutant protein in cell lysates.

To characterize cleavage at Asp964 (which separates EGF-like repeats 24 and 25) in a purified system, we cloned complementary DNA (cDNA) sequences of NOTCH3 corresponding to EGF-like repeats 23 to 26 as fusions to the C terminus of mouse Fc. These recombinant clones were used to make stable 293 cell lines that secreted Fc-NOTCH3(23–26) protein into the conditioned media, from which pure recombinant NOTCH3 fragments were then prepared by Protein A-agarose affinity purification. Since Asp964 is located in the middle of the NOTCH3 sequences of Fc-NOTCH3(23–26), cleavage of the protein at Asp964 is predicted to liberate an Fc-containing fragment that is 8 kDa smaller than the intact protein. In the event of cleavage, the fragmented protein, but not the parent protein, is expected to react with 69B on immunoblots (Fig. 3*B*).

Purified protein Fc-NOTCH3(23–26) from cell lines was separated by SDS-PAGE and demonstrated a major band reactive with anti-Fc antibodies at the expected size for uncleaved protein (Fig 3*C*; Fc band in *red*). This band was not reactive with 69B. But using 69B, we readily detected a smaller band at the size expected after Asp964 cleavage, which increased with time of incubation at 37 °C (Fig. 3*C*).

Cleavage of NOTCH3 at other Asp-Pro sequences (positions 80–81 and 121–122) were shown to be enhanced by acidic pH (18, 19). We incubated Fc-NOTCH3(23–26) at five different pH values and analyzed the protein by immunoblotting using Fc and 69B to detect cleavage at Asp964. As shown in Figure 3, *D* and *A*, truncated 69B-reactive band was observed at all pH values, but there was significantly less at pH 9 (when considering the ratio of 69B-reactive protein to Fc-reactive undigested protein). Moderately acidic conditions (that previously enhanced cleavage at Asp-Pro positions 80–81 and 121–122 (18, 19)) did not enhance cleavage at Asp964, compared with neutral pH (Fig. 3*D*).

Similarly, cleavage of NOTCH3 at Asp-Pro positions 80 to 81 and 121 to 122 is enhanced by protein reduction (18, 19). To test whether the same holds true for cleavage at Asp964, we incubated Fc-NOTCH3(23–26) with increasing doses of TCEP, which resulted in increased cleavage product when assessed by immunoblotting with 69B (Fig. 3*E*; *far right panel*). The reduction-sensitive cutting process was not affected by pretreatment with chaotropic agent guanidinium or with heat treatment, which did not reduce the amount of cleavage facilitated by TCEP (Fig. 3*F*; *first and second sets of proteins*).

To test if Pro965 of the Asp-Pro cleavage sequence is required for cleavage, we created a series of point mutants of Fc-NOTCH3(23–26) in which NOTCH3 residue 965 was mutated to all other amino acids (Fig. 3*G*). Mutant constructs were transiently transfected into 293 cells, and Fc proteins from conditioned media were concentrated using Protein A beads before analysis by immunoblotting. Comparable amounts of Fc protein were recovered from the wildtype construct and all point mutants. As shown in Figure 3, *H*–*J*, the amount of cleaved protein that was reactive to 69B was highest for the wildtype protein containing the wildtype proline residue at position 965. Only trace amounts of 69B-reactive protein were found in all the mutants. This indicates that the proline that follows the Asp964 cleavage site is critical for protein fragmentation.

### Localization of Asp964 cleavage of NOTCH3 in CADASIL brain

**Figure 4:**
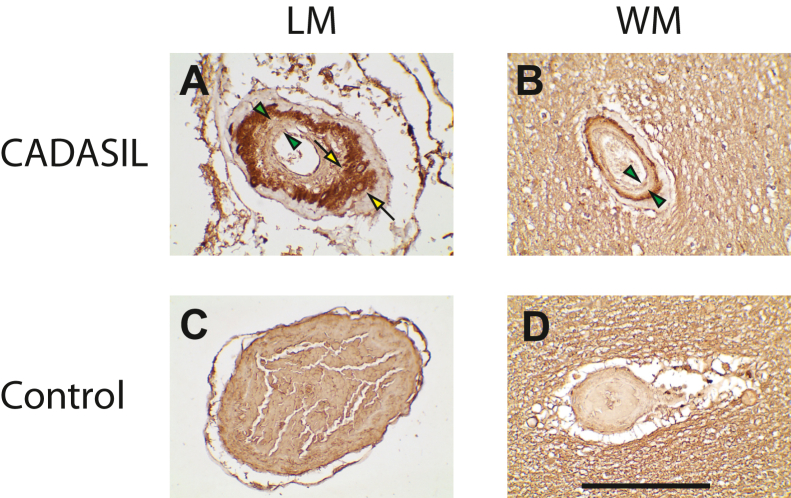
**Distribution of NOTCH3 Asp964 cleavage product in CADASIL.** A series of genetically defined CADASIL brains were examined by immunohistochemistry using 69B. Representative images from frontal lobe leptomeninges (LM; *A* and *C*) and white matter (WM; *B* and *D*) are shown from CADASIL and control brains. Immunoreactivity was consistently present in CADASIL samples but not in controls in both LM and WM. Reactive material was deposited predominantly in arteries like those featured here that displayed significant intimal hyperplasia (*arrowheads*) and medial degeneration (*arrows*). The scale bars represent 100 microns. CADASIL, cerebral autosomal dominant arteriopathy with subcortical infarcts and leukoencephalopathy.

We used immunohistochemistry to analyze the location of Asp964 within arteries in CADASIL brain samples (Fig. 4). When 69B was used on genetically defined CADASIL frontal lobe sections, deposits were found in both leptomeningeal and penetrating vessels of the white matter, particularly in thickened arteries in which vessels showed vascular media degeneration and intimal hyperplasia (regions between *arrows* and *arrowheads*; best seen in leptomeningeal arteries). In comparison, non-CADASIL control brains did not show consistent staining with 69B in cerebral arteries. The reactivity pattern for Asp964 cutting was similar to what we have previously described using antibodies that detect Asp80 and Asp121 cleavage (18, 19). Results represent analysis of 26 genetically defined CADASIL brains and 15 control brains without clinically apparent neurological disease.

**Figure 5:**
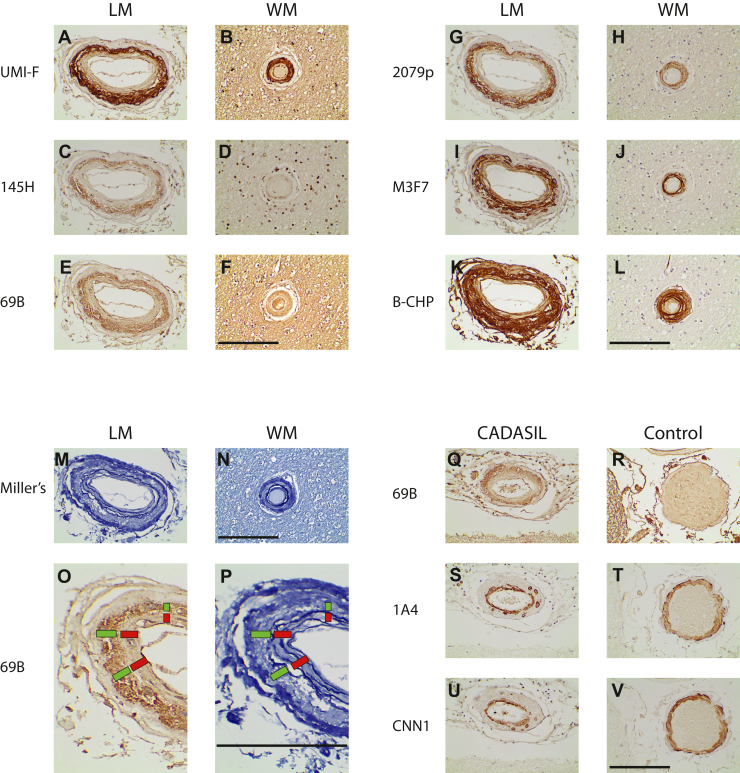
**Localization of NOTCH3 Asp964 cleavage product in relation to other proteins that accumulate in CADASIL.** Serial sections from the same tissue block of CADASIL frontal lobe were stained with probes as shown (*A*–*N*). Probes include UMI-F (for NTF, the product of Asp80 cleavage; (18)), 145H (for NTF2, the product of Asp121 cleavage; (19)); 69B (for cleavage at Asp964). 2079p (purified polyclonal sera 2079 against reduced conformation of NOTCH3; (17)), M3F7 (for type IV collagen; (28)), B-CHP (pan-collagen binding peptide; (29)), and Miller’s stain for elastin (19). Both leptomeningeal (LM) and white matter (WM) vessels showed similar distributions, but the localization was clearer in LM arteries because of their size and clarity of the internal elastic lamina on Miller’s staining. We enlarged the images of the 69B staining of the LM artery (*O*) and Miller’s staining of the same artery (*P*) to provide better visualization of the subregions of the artery. The *red bar* indicates thickened intima that contains fragmented internal elastic lamina. *Q*–*V*, serial sections of CADASIL (*Q*, *S*, and *U*) and control (*R*, *T*, and *V*) leptomeningeal arteries were analyzed by 69B (*Q* and *R*) and 1A4 (for ACTA2; *S* and *T*) and CNN1 (*U* and *V*) antibodies. The *green bar* indicates the vascular media, which are most strongly stained with 69B. The scale bars represent 100 microns. CADASIL, cerebral autosomal dominant arteriopathy with subcortical infarcts and leukoencephalopathy.

To better define the location of Asp964 cleavage in CADASIL samples, we stained serial sections with 69B (for Asp964 cleavage) and with antibodies UMI-F and 145H (for proteolysis at Asp80 and Asp121; Fig. 5). This revealed a markedly similar pattern in leptomeningeal arteries, where the borders between vascular layers are readily defined. Staining with all the antibodies appeared most intensely in the vascular media, defined by regions outside the internal elastic lamina that is seen in Miller staining of adjacent brain sections (Fig. 5; *blue stain*). The Asp964 cleavage was not as extensive as collagen deposition, which was found in both media and intima (Fig. 5; B-CHP for all collagens and M3F7 for type IV collagen). The 69B immunoreactivity also colocalized well with 2079 (NOTCH3 conformational antibody; (17)) in serial sections of the same artery (Fig. 5).

Serial sections were also stained with 69B and mature smooth muscle markers ACTA2 and CNN1 (Fig. 5). In CADASIL, 69B reactivity in leptomeningeal arteries did not completely coincide with either smooth muscle marker; both ACTA2 and CNN1 was expressed strongly in the intima and in remnant balloon cells of the media. This agreed with prior work in which we showed redistribution of smooth muscle markers to the intima, whereas NOTCH3 remained localized to the media without preference to viable cells (20). In control arteries, 69B reactivity was not appreciated above background, whereas ACTA2 and CNN1 were expressed strongly in the media.

### Identification of proteins that interact with Asp964-cleaved NOTCH3 fragmentation product

Numerous proteins interact with NOTCH3 and have been considered potential modulators of CADASIL pathogenesis (7, 8, 9, 10, 11, 21, 22, 23). Having identified Asp964 cleavage in the vascular media of CADASIL, the principal site of pathology, we next explored whether the proteolytic product of this cleavage could interact with other proteins; moreover, we sought to test on a proteome-wide basis if some proteins preferentially interact with the cleavage product over the intact NOTCH3 protein.

**Figure 6:**
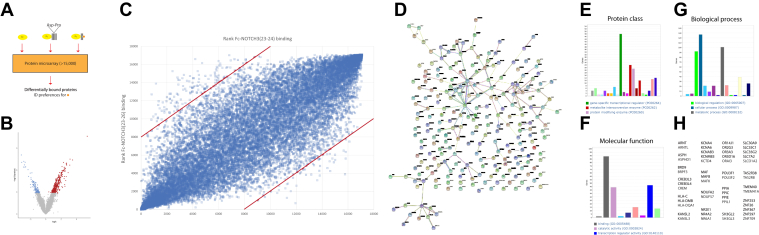
**Identification of proteins that bind to NOTCH3 protein after cleavage at Asp964.***A*, outline of experimental approach. Protein microarrays (CDI; (31)) with over 15,000 spotted purified proteins were exposed to purified Fc, Fc fused to NOTCH3 EGF 23 to 26 (Fc-NOTCH3(23–26)), or Fc fused to NOTCH3 EGF23 to 24 (Fc-NOTCH3(23–24)). The latter protein terminates in Asp964, which is generated by Asp-Pro cleavage shown. *B*, proteins exhibiting preferential binding to Asp964-terminated probe are depicted in a volcano plot that compares the ratio of signal of Fc-NOTCH3(23–24) to Fc-NOTCH3(23–26). The fold change for each protein shown on the *x*-axis; the *p* value is shown on the *y*-axis. *Red and blue spots* represent proteins with higher binding to Fc-NOTCH3(23–24) (protein terminating in Asp964) and Fc-NOTCH3(23–26), respectively. *C*, rank-order plot. Each protein intensity was rank ordered for each probe with low rank number corresponding to higher signal. The rank order for binding to Fc-NOTCH3(23–24) (terminating in Asp964) and for Fc-NOTCH3(23–26) is on the *x*-axis and *y*-axis, respectively. The *left upper quadrant* includes proteins with preferential binding to Fc-NOTCH3(23–24) (terminating in Asp964). *D*, protein relationship and interaction analysis was performing using STRING (August 2022) for molecules displaying increased binding to Fc-NOTCH3(23–24) (terminating in Asp964). *E* and *F*, analysis of protein classes, biological processes, and molecular function of proteins with increased binding to Fc-NOTCH3(23–24) (terminating in Asp964), as determined by Gene Ontogeny classifications using PANTHER (version from August 2022). The *top* three categories are listed below the histograms. Data with legends showing the complete set of categories for each of these analyses are included in the supporting information. *H*, shows proteins that were members of groups of related molecules that showed increased binding to Fc-NOTCH3(23–24) (terminating in Asp964).

We applied purified protein fragments of Fc-NOTCH3(23–24), which includes two NOTCH3 EGF-like modules that terminate with Asp964 to protein microarrays that contain over 15,000 purified human proteins. A comparison group was generated by probing parallel protein microarrays with Fc-NOTCH3(23–26), in which four EGF-like modules include the uncleaved Asp-Pro sequence (Fig. 6*A*).

Binding signals for all proteins on these microarrays revealed satisfactory reproducibility of binding levels. The experiment revealed that a subset of proteins were preferentially bound by Fc-NOTCH3(23–24) compared with Fc-NOTCH3(23–26). Another subset of proteins preferred binding to Fc-NOTCH3(23–26). An overview of the aggregate data is shown in Figure 6*B*, which shows a conventional volcano plot of the results, and in Figure 6*C*, we show a dot plot of all proteins tested by rank order of signal intensity.

To explore whether individual proteins that bind to Asp964 could be classified according to biological pathway, we performed several analyses using established algorithms; a total of 261 proteins exhibited 1.5-fold increased binding to Fc-NOTCH3(23–24) compared with Fc-NOTCH3(23–26) (see Supporting information). The STRING algorithm (https://string-db.org) for exploration of protein interactions (Fig. 6*D*) shows potential interactions between proteins that bind to cleavage products; notable nodes in these networks include MAPK1 and PRKACG. Class analysis using the PANTHER algorithm (www.pantherdb.org) noted that the main protein class was transcriptional activators and metabolic enzymes (Fig. 6*E*). Binding proteins and cellular process (Fig. 6, *F* and *G*) were the most common categorizations of NOTCH3 truncation product–binding proteins. Of note, 18 groups of related proteins were identified as having preference for binding to protein terminating in Asp964 (Fig. 6*H*), which suggests that this NOTCH3 fragment may target a homologous protein domain common to each member of the class. On the other hand, most of these classes do not have significant homology, indicating that the targets of the Asp964-terminated NOTCH3 fragment are heterogeneous.

### Regulation of cleavage at Asp964

Prior work has established experimental factors that regulated NOTCH3 Asp-Pro cleavage (18, 19). To assess the effects of these factors on cleavage at Asp964, we analyzed purified Fc-NOTCH3(23–26) under defined conditions *in vitro*, using 69B immunoblotting to detect and quantify the efficiency of Asp964 proteolysis.

**Figure 7:**
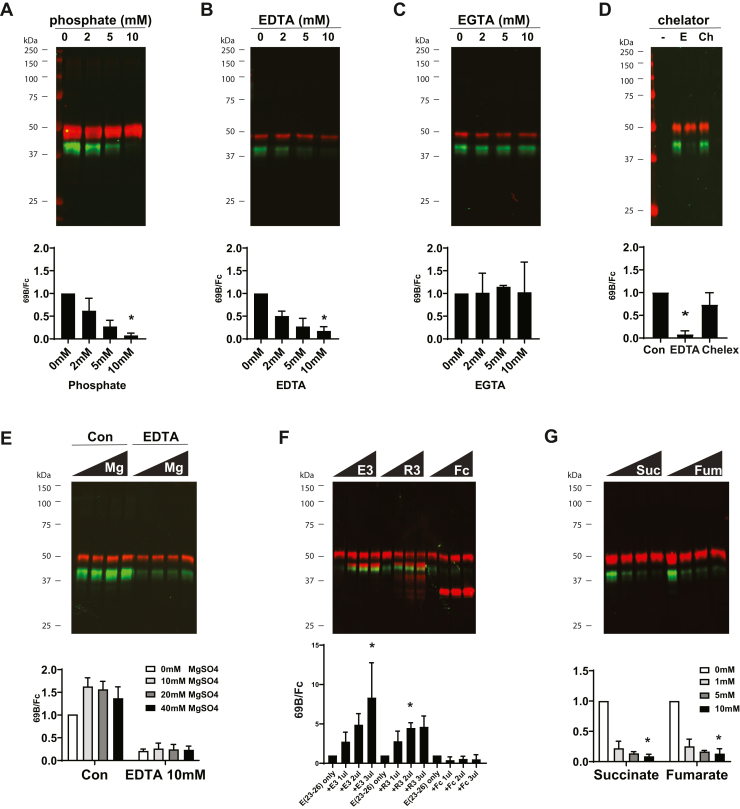
**Determination of factors that modulate nonenzymatic cleavage of NOTCH3 at Asp964.***A*–*C*, purified recombinant Fc-NOTCH3(23–26) was diluted in water supplemented with additives as noted and then incubated at 37 °C for 1 h as described for Figure 3. Reaction products were resolved by SDS-PAGE, and immunoblots were probed with 69B for Asp964 cleavage and Fc for undigested protein. Asp964 cleavage products were quantified as for Figure 3*D*. Fc-NOTCH3(23–26) proteins were diluted without treatment (Con), treated with 10 mM EDTA, or treated with 10 μl Chelex beads at pH 7.0 (0.1 g beads rinsed with 1 ml 0.5 N sodium acetate buffers, followed by five times washing by 1 ml distilled water) prior to incubation for 1 h at 37 °C and then analysis was done for Asp964 cleavage. Final reaction volumes were 20 μl. *E*, Fc-NOTCH3(23–26) proteins were treated with combinations of water only (Con) or 10 mM EDTA and also supplemented with MgSO_4_ at concentrations shown prior to quantification for cleavage at Asp964. *F*, Fc-NOTCH3(23–26) protein [E(23–26)] was supplemented with increasing amounts of proteins indicated prior to dilution and incubation at 37 °C. E3 = Fc-NOTCH3(1–3). R3 = Fc-NOTCH3(1–3) with the R90C mutations. Fc is mouse Fc protein alone. All protein stocks were 200 ng/μl. *G*, Fc-NOTCH3(23–26) proteins were mixed with water and potentially inhibitory compounds at concentrations indicated. Quantification of cleavage was performed as in (*A*–*C*). Additional tests for inhibition were performed using additional compounds, shown in Fig. S1. All experiments were done at least three times. Merged pseudocolored images are shown, with *green* for 69B and *red* for Fc. ∗ indicates differences between group with a *p* < 0.05.

In accordance with prior studies (18, 19), specific ions had effects on proteolysis at Asp964. With increasing phosphate ion concentrations, cleavage of Fc-NOTCH3(23–26) decreased steadily, as determined by quantification of the ratio of cleaved product to total Fc protein (Fig.7*A*). Similarly, treatment of Fc-NOTCH3(23–26) with EDTA, but not EGTA, resulted in inhibition of cleavage at Asp964 (Fig. 7, *B* and *C*). The treatment was not related to chelation properties because incubation with Chelex resin failed to block the cleavage of NOTCH3 (Fig. 7*D*). Moreover, addition of magnesium ion to cleavage reactions did not neutralize the inhibitory effect of EDTA (Fig. 7*E*).

Finally, because Asp-Pro cleavage of NOTCH3 at Asp80 and Asp121 (18, 19) are increased with addition of NOTCH3 protein, we conducted studies to investigate the effect of addition of NOTCH3 protein on cleavage reactions. When Fc-NOTCH3(1–3) fusion protein (13) was included in the cleavage reaction, we noted that the cleavage was significantly increased (Fig. 7*F*; E3 signifies Fc-NOTCH(1–3)), as we have observed for other NOTCH3 Asp-Pro reactions. There was no additional enhancement of cleavage with CADASIL mutant protein (Fig. 7*F*; R3 that contains the R90C mutation). Fc by itself had no effect on cleavage at Asp964.

We tested a panel of dicarboxylic acids for their effects on cleavage at Asp964. Of those tested, we found that succinate and fumarate significantly inhibited cleavage (Fig. 7*G*). The following compounds were not found to affect the cleavage reaction: itaconic acid, citrate, l-glutamic acid, d-glutamic acid, l-aspartic acid, d-aspartic acid, *N*-methyl-d-aspartic acid, triethylene-tetramine. Data are shown in Fig. S1.

## Discussion

These studies demonstrate that a third Asp-Pro sequence at Asp964 in NOTCH3 is susceptible to cleavage in CADASIL. This cleavage event demonstrates similarities to cleavage at Asp80 and Asp121 in the vascular distribution and chemical properties. In addition, using large-scale protein microarrays, we show that cleavage alters interaction partners of NOTCH3 protein.

### New Asp-Pro cleavage in small vessel disease protein NOTCH3 implicates extensive protein stress–related instability

CADASIL is a disorder marked by stereotyped mutations in *NOTCH3*, which alter disulfide bonding. As such, it has been suspected that NOTCH3 protein conformational changes are key events in disease pathogenesis. The Asp964 cleavage event is similar to the previously described Asp80 and Asp121 in its occurrence in CADASIL and its regulation by chemical reduction, an *in vitro* stressor that simulates conformational changes caused by cysteine mutations. The activation of cleavage by reducing agents for all sites is relevant to CADASIL, in which the reduction of protein by virtue of cysteine mutations has been suspected (13, 17).

These findings prompt other questions: Are there other Asp-Pro sequences in NOTCH3 that are regulated by mutations or reductive stress? Since proteins can be transreduced by NOTCH3 fragments released by Asp-Pro cleavage, are additional vascular proteins that undergo Asp-Pro cleavage in small vessel disease? Extension of Asp-Pro to other disorders of the vascular system (*e.g.*, atherosclerosis or coronary artery atherosclerosis) would, in addition, suggest that Asp-Pro cleavage may play a more general role in cerebrovascular pathology.

### Common chemical characteristics link three independent cleavage sites of NOTCH3

Asp-Pro cleavage has been described now at three sites in NOTCH3 and in at least three additional proteins (24, 25, 26); in each case, the proteins are cleaved without the addition of additional proteins, which supports a nonenzymatic mechanism of action. Because our current study shows that the chemical enhancement and inhibition of cleavage at Asp964 is similar to other NOTCH3 sites, a similar mechanism of action for all three Asp-Pro sites seems probable. Of note, however, the cleavage at Asp964 is different from other sites in that it does not increase under neutral *versus* acidic conditions and that denaturation of the protein does not effectively block cleavage.

We extend the profiling of Asp-Pro cleavage by demonstrating that additional dicarboxylate ions are capable of inhibition of this process. Although a single structure did not stand out as an exceptionally potent inhibitor of Asp964 cleavage, the sensitivity to phosphate and dicarboxylates suggests that metabolic alterations that influence the concentration of small molecules in the vessel wall may participate in regulating the concentrations of Asp964 formation.

### Cleavage may result in liberation of new protein-binding motifs within the site of disease

To identify protein-binding partners to Asp964 cleaved NOTCH3 on a proteome-wide basis, we used a high-throughput protein microarray and compared binding levels of cleaved NOTCH3 to uncleaved NOTCH3. A significant number of proteins were found that bound more avidly to the cleaved protein. The change in affinity of multiple binding partners could alter the interactome of proteins in the arterial extracellular matrix by either competing with other binding interactions or by creating new pairs of proteins with altered biological function. The increase in binding may also contribute to protein aggregation cascades and participate in the massive buildup of proteins found in CADASIL vessels. Of note, many of the partners identified on microarray analysis are cytoplasmic proteins, which may only interact with NOTCH3 upon cell lysis, a process of unclear significance in early pathogenesis of disease. Future work will be required to understand the downstream consequences of the new protein interactions with Asp964.

The existence of multiple new protein interaction partners with Asp964 indicates that the cleaved protein is distinct in conformation relative to the uncleaved protein, a process that may be due to the release of steric hindrance or by the availability of a new C-terminal epitope. Similar studies on other cleaved products of NOTCH3 will be required to disclose the full extent of consequences of the multiple NOTCH3 protein cleavage in CADASIL. Of note, several subunits of voltage-gated potassium channels were found to be partners of the cleaved NOTCH3 fragment ending at Asp964; the significance of this needs to be pursued as Nelson *et al.* (27) have shown that in a mouse model of CADASIL, stimulus-induced potassium channel function is attenuated in vessels.

One limitation of this study is that we have been unable to detect the cleaved product by Western blotting. It is not clear whether the protein is further modified, rendering it unavailable on blots. In addition, only a small fraction of NOTCH3 is cleavable at Asp964 (judged by the high amount of residual uncut proteins in cleavage assays in Figs. 3 and 7). Consequently, more sensitive methods will be needed to quantify cleavage in tissue extracts where only a fraction of the cells express NOTCH3.

In summary, we describe a third Asp-Pro site at Asp964 in NOTCH3 that is cleaved in CADASIL arteries. The fragmentation, which can occur by nonenzymatic means, is blocked by small polyanionic compounds and results in a NOTCH3 product whose C terminus binds to a diverse family of proteins that do not interact with the uncleaved NOTCH3 protein. These findings suggest specific additional work to uncover protein interaction networks altered in small vessel disease.

## Experimental procedures

### Antibodies

The 69B rabbit monoclonal antibody was generated for this study using procedures that are detailed elsewhere (19). All animal experiments were performed by GenScript and reviewed by the GenScript Institutional Animal Care and Use Committee (animal protocol no.: ANT19-005). Experiments were performed in accordance with ARRIVE (Animal Research: Reporting of In Vivo Experiments) guidelines (except that a control group of nonpeptide immunized animals was not included to reduce unnecessary animal use). All experiments were performed in accordance with relevant guidelines and regulations; specifically, methods of euthanasia were consistent with the recommendations of the Panel on Euthanasia of the American Veterinary Medical Association.

The peptide antigen SCLCRPGYTGAHCQHEAD (terminating in human NOTCH3 residue 964) was synthesized and used for immunization of New Zealand rabbits. Antibody cDNAs from spleen cells isolated from animals were transfected to generate intact antibodies. These antibodies were then used to identify candidate antigen-reactive clones. The clone 69B was used for all experiments because it demonstrated specificity for the neoepitope predicted to result from cleavage after Asp964.

Antibodies UMI-D and UMI-F (18) and 2079p (17) were previously used for immunohistochemical (IHC) staining. Recombinant antibodies (19) were generated by PolyJet (SignaGen) transient transfection of paired heavy- and light-chain cDNA expression clones into 293 cells grown in T75 flasks. After a day, media were replaced with OptiMEM (Invitrogen) and grown for another 3 days. Undiluted conditioned media were used for staining. M3F7 (Developmental Studies Hybridoma Bank at the University of Iowa; catalog no.: M3F7, supernatant; used at 1 μg/ml; (28)) was used for type IV collagen. The collagen-binding peptide B-CHP (3Helix; catalog no.: B-CHP, biotin conjugate; 50 μM; (29)) was used to detect conformationally altered collagen proteins by IHC. GFP antibody (B-2; catalog no.: sc-9996; Santa Cruz) was used for immunoblots (1:1000 dilution) and for immunocytochemistry (1:200 dilution). N-terminal NOTCH3 detection by immunoblotting utilized the M01 mouse monoclonal antibody (Abnova) that recognizes a sequence in the first EGF repeat.

### Tissue and cell staining

Formalin-fixed sections were obtained from autopsies of CADASIL patients with NOTCH3 cysteine-altering mutations (10, 17, 20, 30). Control samples were obtained from the Alzheimer’s Disease Center at the University of Michigan and the Brain Bank of the National Institute for Developmental and Childhood Disorders at the University of Maryland (30). All samples were from medial frontal lobe. Five micron sections underwent IHC staining after antigen retrieval in citrate buffer; sections were blocked (2% bovine serum albumin in PBS) for 30 min; primary antibody was then added overnight in a humidified chamber at room temperature. Biotinylated secondary antibodies in blocking solution at 1:200 dilution were used for 30 min followed by 15 min incubation of ABC solution (Vectastain Elite ABC kit; Vector Lab; catalog no.: NC9293436). Finally, chromogenic detection by 3,3′-diaminobenzidine was executed by applying ImmPACT 3,3′-diaminobenzidine horseradish peroxidase substrate for 1 to 5 min (Vector Lab; catalog no.: NC9567138); all washes were done with running tap water (three to five exchanges); after IHC, hematoxylin counterstaining was conducted. Tissue antigen integrity was confirmed by stains for BRIC231, which recognizes human endothelial cells (anti-H; Santa Cruz; catalog no.: sc-59467, 200 μg/ml). Use of antibodies to smooth muscle actin (1A4; Sigma) and CNN1 (polyclonal; Sigma) have been described before (20). Immunocytochemistry was performed as described before (19) on transfected 293 cells that were fixed with formalin. GFP antibodies were used as positive controls for protein expression.

B-CHP staining was performed as described by the manufacturer (50 μM; 3Helix; (29)). Essentially, the same procedure as for IHC was conducted, except that the secondary antibody step was deleted. The probe was heated before application. All samples underwent heat-mediated antigen retrieval, a condition that denatures all collagens and results in B-CHP binding to all collagen in the tissue. Miller’s stain (EMS 2607-05), for elastic fibers, was used undiluted on sections adjacent to immunostained samples (19).

### DNA constructs

PCR-generated fragments or synthesized DNA were inserted C-terminal to the enhanced GFP open reading frame of pEGFP-C3 (Clontech) by standard ligation procedures (19). Clones were sequenced to confirm the presence of open reading frames, which included pieces of NOTCH3 of different lengths with and without point mutations. The Fc-E(x) constructs were generated in pSecTag (Invitrogen), which includes an immunoglobulin gene signal sequence. The Fc clones (19) include mouse immunoglobulin G Fc fused at its C terminus to EGF domains (x). For example, Fc-E(23–26) includes EGF domains 23, 24, 25, and 26, and Fc-E(23–24) includes domains 23 and 24, ending in Asp964. We performed standard nested PCR with mutant oligonucleotides to generate point mutations in clones.

### Protein interaction array analysis

Purified human proteins printed on slides without denaturation (glutathione-*S*-transferase linked and produced in yeast; HuProt arrays from CDI; (31)) were probed with three Fc proteins at 2.0 μg/ml at 4 °C overnight in Tris-buffered saline with Tween-20 (TBST). Purified Fc protein was purchased from R&D Systems. Recombinant Fc-E(23–26) and Fc-E(23–24) were produced and purified as described later. Fluorescent secondary antibodies (Cy3-antimouse immunoglobulin G secondary) were used to detect labeled positions of the microarrays. Experiments were performed in duplicate. *t* Test was used to compare Fc-E(23–26)- and Fc-E(23–24)-generated signals, with proteins of interest targeted for *p* < 0.05 and signal changes 1.5 greater in the Fc-E(23–24) arrays. Nonspecific hits that were generated by secondary antibodies alone or Fc alone were discarded. Duplicates of each protein probing showed reproducibility of R2 + 0.9915 and 0.9872 for Fc-E(23–26) and Fc-E(23–24), respectively.

Analysis was done by generating a conventional volcano plot that arrayed each protein by fold change and *p* value. A second analysis was generated by rank ordering all proteins by signal generated by a specific probe; the highest signal was used for each protein represented by multiple positions in the array.

### Recombinant protein preparation and analysis of proteolysis

Human embryonic kidney 293 cells grown to 90 to 100% confluence were transfected using PolyJet (SignaGen; catalog no.: SL100688) per the manufacturer's protocol (19). Fc-E(23–26) plasmids were combined with vectors that encode puromycin resistance, and the transfected cells were selected for resistance by supplementing growth media with puromycin; cell colonies were individually selected and independently propagated to select high-expressing clones. The conditioned media of these clones (in OptiMEM; Invitrogen; catalog no.: 31985070) were collected, and multiple fractions were combined and purified by protein A agarose chromatography (15). Acidic elution of Fc protein was followed by immediate neutralization with neutral Tris; finally, protein was dialyzed with two exchanges of PBS. The purity of the Fc-recombinant preparations was determined using gel electrophoresis and silver staining.

For proteolysis studies, 200 ng of purified proteins (at 200 ng/μl, unless specified) was diluted in water and supplemented with chemicals described in each experiment (19). Except where indicated, the diluted protein was incubated at 37 °C for 1 h followed by Western blot analysis using 69B to detect cleavage of protein. Cleaved protein signal was normalized to total Fc signal from the same lane of each immunoblot.

For protein stimulation studies, Fc-NOTCH(23–26) was supplemented with Fc-NOTCH3(1–3) and mutants, which have been described before (13, 15).

### Western and dot blotting

We have previously described immunoblotting and dot blotting (19). In brief, proteins were run on SDS polyacrylamide gels and transferred to nitrocellulose in an iBlot 2 instrument (Invitrogen; method P0 20V for 1 min/23V for 4 min/25V for 2 min). Membranes were blocked in TBST supplemented with 5% milk and then probed with primary antibodies in TBST at 4 ^°^C overnight. Fluorescently labeled secondary antibodies in TBST at room temperature were used for 30 min. Wash steps after each antibody incubation were performed thrice at room temperature using TBST. Secondary antibody used included: donkey antimouse IRDye 680RD (Li-Cor; catalog no.: 926-68072, 1:10,000 dilution, AB_10953628) and goat anti-rabbit IRDye 800CW (Li-Cor; catalog no.: 926-32211, 1:10,000 dilution, AB_2651127). Dot blots were performed on spotted peptides (sequences specified in Fig. 2; synthesized by GenScript). Peptides (1 μg/μl) in 1 μl of water were spotted on membranes and allowed to dry at room temperature (19). Membranes were blocked in TBST supplemented with 5% milk and then probed with primary and secondary antibodies as described previously for Western blots. For immunoblots and dot blots, we detected and quantified signal using a Li-Cor Odyssey Imager for blot imaging with detection settings at 700 and 800 nm and Li-Core Image Studio software for data capture and quantification.

### Statistics

Mann–Whitney U tests were used to determine the differences between two experimental groups. Kruskal–Wallis tests followed by Dunn multiple comparison post hoc analysis were used to compare the differences among three or more experimental groups (Prism 8 analysis software; GraphPad). A probability value of <0.05 was considered statistically significant.

## Data availability

Experimental data from proteomic arrays are available upon request. NOTCH3 cDNA plasmids generated for this study are available on request.

## Supporting information

This article contains supporting information.

## Conflict of interest

The authors declare that they have no conflicts of interest with the contents of this article.
