# Supplementary material for: A midposition NOTCH3 truncation in inherited cerebral small vessel disease may affect the protein interactome
Source: J Biol Chem. 2022 Dec 5;299(1):102772. doi: 10.1016/j.jbc.2022.102772 (PMC9808000; doi:10.1016/j.jbc.2022.102772)
Supplement: Supplemental information [file mmc1.pdf]

Supplemental Information for:

A mid-position NOTCH3 truncation in inherited cerebral small vessel disease may affect the protein interactome

Soo Jung Lee [1,3] *, Xiaojie Zhang [1,3] *, and Michael M. Wang [1,2,3] ^

*Contributed equally

[1]Department of Neurology and [2]Molecular and Integrative Physiology, University of Michigan, Ann Arbor, MI

[3]Neurology Service, VA Ann Arbor Healthcare System, Ann Arbor, MI

^ Corresponding Author

2215 Fuller Rd, Building 31-221, Ann Arbor, MI 48105

734-845-5202

micwang@umich.edu

Running title: NOTCH3 truncation at mid-position Asp-Pro bond

Keywords: NOTCH3, CADASIL, Asp-Pro bond, non-enzymatic cleavage, protein interactions

Proteins exhibiting 1.5-fold increased binding Fc-NOTCH3(23-24) compared to Fc-NOTCH3(23-26) proteins on protein microarrays n=261.

PPIL1

MAPK1

MRPL12

CRK

SORBS3

PPIC

BIN1

PPIE

BIN1

HNRNPUL1

STAM

MEIS2

EID1

BIN1

CCDC102B

NAPB

DBNL

VCX3A

TNFSF14

HCLS1

MFRP

Bsh

DBNL

NDOR1

MSRB1

MYLK

ZNF709

LYPD5

OTX2

HLA-DMB

EGR4

SLCO1A2

ST18

PYGM

ZNF26

OTULIN

PPIA

GPM6A

GPN2

POLR3K

KLHL1

CREB3L3

PRKACG

ZNF367

TF

NDUFS7

JDP2

UBE2G2

CASC2

BRD9

RAD23A

PKNOX2

MPG

NANOGP8

HMSD

JHU11996

HIST1H3A

MLX

SH3GL2

KRT79

POU3F2

HMGA1

DPCR1

ERG

AMOT

AGO2

DMTF1

UTP18

YPEL2

SNRPA

GNG11

VSX1

MAFB

MAN2B2

ZBTB48

HMX2

ASPHD1

FBXW5

BHLHE41

SMC6

GBE1

CREB3L4

MAMDC2

STAT2

NACC2

WFS1

SH3GL3

MAF

CENPM

FARP1

CELA3A

ENPP5

BRPF3

C19orf54

NPL

CES1

BNC2

ABHD13

RERGL

TAS2R8

NR2E1

CA14

LTB

C19orf18

KCNA6

LGALS14

PDLIM5

C4orf32

ISL1

ARNT

KRTAP10-10

IL1RL1

ZNF597

SLC35C1

SLC30A9

BEND5

HIC2

Gsc2

KCNA4

LY9

G3XAM8

OR3A3

KDR

STK31

GJA8

PSMA2

IL18

PCGF3

KANSL2

IRX1

NDST1

HOXB7

TMEM41A

LIPF

SMR3A

MNT

Pou3f1

ATF6B

DLEU1

Lupus La

RINT1

KCNMB3

RABEPK

ORAI3

SNAI1

CREM

AREL1

PLA2G7

ASAP3

ADAM21

DCAKD

NPY4R

PDZD4

MC5R

HLA-C

KJ901395

KCNAB3

NR6A1

MKNK1

GLRB

HG507164.1

LALBA

EXOSC1

PRRG1

MRGPRF

APOBEC3B

TOM1L1

ZER1

PSMC4

SLC7A2

NR4A2

NUDCD1

SCRT2

HHEX

NOX1

CLEC2B

PPA2

ABCG1

NET1

HLA-DQA1

GMPR2

LINC01315

SLC35G2

NDUFA2

ARNTL

RNF185

CHIT1

OR5D16

Ehf

MAFK

SENP6

CPXM1

PCDH1

EIF4ENIF1

TNIP1

NANOGP8

GNAS

HHLA3

TUBA8

LIMK1

NOL4

PRKCI

CYP4F12

ZCCHC2

MEPCE

KCTD4

NOSTRIN

TSPAN17

ASPH

OR14J1

DKK3

IFNA1

OTULIN

FANCL

VGLL2

RHOD

SPAG11B

ZSCAN2

CPO

CAV2

COMMD4

TMEM40

KANSL3

ADCK4

RPL12

GCH1

ZNF253

OR2G3

DMRTA2

SLFNL1

ZEB2

SARNP

TYMS

Q14591-2

PARS2

NKX2-3

CNEP1R1

TAS2R38

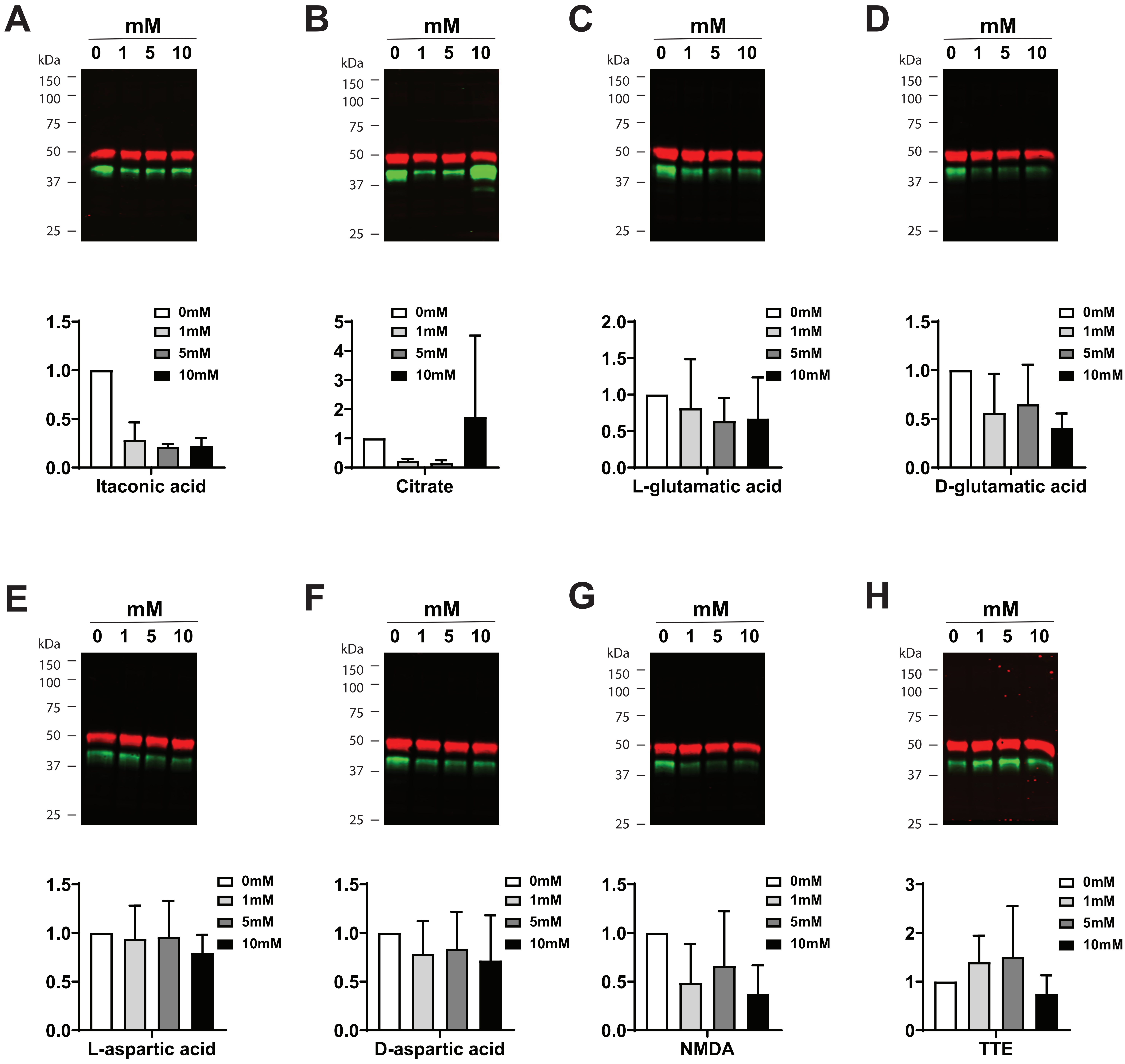

Supplementary Fig 1. Factors that modulate non-enzymatic cleavage of NOTCH3 at Asp964. Purified recombinant Fc-NOTCH3(23-26) was diluted in water supplemented with additives as noted and then incubated at 37C for one hour as described in Figure 3. Reaction products were resolved by SDS-PAGE and immunoblots probed with 69B for Asp964 cleavage and Fc for undigested protein. Asp964 cleavage products were quantified as in Figure 3. All experiments were done at least three times. Merged, pseudocolored images are shown, with green for 69B and red for Fc. The p values for differences between negative controls and 10mM concentrations of test substances are shown below.

Dicarboxylic acids and diamines (10mM) and effect on inhibition of NOTCH3 cutting:

**Chemicals**	**Kruskal-wallis test p value**
Succinate	0.0129
Fumarate	0.0198
Itaconic acid	0.0747
Citrate	0.2684
L-glutamic acid	0.2366
D-glutamic acid	0.1852
L-aspartic acid	0.4289
D-aspartic acid	0.7891
N-methyl-D-aspartic acid (NMDA)	0.1739
Triethylene-tetramine(TTE)	0.5905
